# Fabrication and Characterization of Gelatin Stabilized Silver Nanoparticles under UV-Light

**DOI:** 10.3390/ijms12096346

**Published:** 2011-09-23

**Authors:** Majid Darroudi, Mansor B. Ahmad, Ali Khorsand Zak, Reza Zamiri, Mohammad Hakimi

**Affiliations:** 1 Department of Chemistry, Universiti Putra Malaysia, UPM Serdang 43400, Selangor, Malaysia; E-Mail: mansorahmad@science.upm.edu.my; 2 Department of Modern Sciences and Technologies, School of Medicine, Mashhad University of Medical Sciences, Mashhad 9177948564, Iran; 3 Low Dimensional Materials Research Center, Department of Physics, Faculty of Science, University of Malaya, Kuala Lumpur 50603, Malaysia; E-Mail: alikhorsandzak@gmail.com; 4 Department of Physics, Universiti Putra Malaysia, UPM Serdang 43400, Selangor, Malaysia; E-Mail: zamiri.r@gmail.com; 5 Chemistry Department, Payame Noor University, Tehran 19395-4697, Iran; E-Mail: mohakimi@yahoo.com

**Keywords:** silver nanoparticles, gelatin, UV-light, stabilizer, UV-Vis spectroscopy

## Abstract

Silver nanoparticles (Ag-NPs) were successfully synthesized using the UV irradiation of aqueous solutions containing AgNO_3_ and gelatin as a silver source and stabilizer, respectively. The UV irradiation times influence the particles’ diameter of the Ag-NPs, as evidenced from surface plasmon resonance (SPR) bands and transmission electron microscopy (TEM) images. When the UV irradiation time was increased, the mean size of particles continuously decreased as a result of photoinduced Ag-NPs fragmentation. Based on X-ray diffraction (XRD), the UV-irradiated Ag-NPs were a face-centered cubic (fcc) single crystal without any impurity. This study reveals that the UV irradiation-mediated method is a green chemistry and promising route for the synthesis of stable Ag-NPs for several applications (e.g., medical and surgical devices). The important advantages of this method are that it is cheap, easy, and free of toxic materials.

## 1. Introduction

In recent years, the preparation of nanosized noble metallic particles has garnered much more attention due to their different properties, such as electric and magnetic properties compared to macro-sized metal phases. As a result, the use of these nanomaterials in industrial and technological applications for various fields is quickly growing [1–3]. Metallic nanoparticles such as Ag-NPs are very important; therefore, many synthetic routes have been explored for their preparation (*i.e.*, physical and chemical methods) [4–7]. These techniques require special apparatuses and fabrication of metallic nanoparticles, which can be very difficult in several cases. Using new and easy methods to prepare metallic nanoparticles is still actually possible. Recently, different methods have been applied to prepare various metallic nanoparticles, especially in size and shape, using light at different wavelengths in solutions of metallic sources in the presence of chemical materials as the stabilizers or size controller [8,9]. Previous studies examining the UV irradiation method for preparing Ag-NPs have used a variety of stabilizers (e.g., polyvinylpyrrolidone (PVP) [10], polyurethane (PU) [11], and poly(methacrylic acid) (PMA) [12,13]); in the current study, we introduce gelatin as a green stabilizer and clearly indicate how the UV irradiation dose can influence the properties of the metallic nanoparticles (e.g., particle size). In previous work, we used stabilizers to prevent particle agglomeration in the fabrication of nanoparticles [14–18]; however, in the current work, we investigated the effect of UV irradiation times on the fabrication of size-controllable Ag-NPs.

## 2. Results and Discussion

In this study, we attempted the fabrication of nanometer silver using the UV irradiation method in which gelatin is used as a green stabilizer. We investigated the effects of UV irradiation times, in addition to Ag^+^, according to the size of the as-synthesized Ag-NPs. We employed gelatin as a capping medium for silver cations and a stabilizing agent for prepared Ag-NPs. After initial preparation of particles via UV irradiation, the prepared particles were dissociated through further UV irradiation to form smaller particles stabilized by the amine pendant groups on the gelatin backbone, ultimately leading to the formation of gelatin-stabilized Ag-NPs [19]. The reduction method normally involves radiolysis of aquatic solutions, which provides an effective route for reducing the metallic ions of transition metals. Using this method, aquatic solutions are exposed to UV-irradiation, and the solvated electrons can be produced. In turn, the produced solvated electrons reduce the metallic cations to the metallic atoms and finally coalesce to form agglomerates, as shortly explained by the following reactions [20]:

(1) $$H_{2}O\overset{h\nu }{\to }e_{aq}^{-}+H_{3}O^{+}+H^{0}+H_{2}+{OH}^{0}+H_{2}O_{2}+\ldots$$

(2) $${Ag}^{+}+e_{aq}^{-}\overset{\text{Reduction}}{\to }{Ag}^{0}$$

(3) $${Ag}^{0}+{Ag}^{+}\to {Ag}_{2}^{+}$$

(4) $${Ag}^{0}+{Ag}_{2}^{+}\to {Ag}_{3}^{+}$$

**Figure 1:**
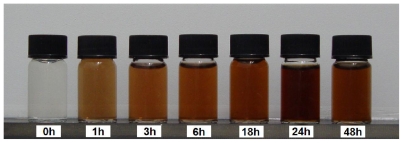
Photograph of synthesized Ag-NPs in gelatin solution (1%) at different UV irradiation times.

The color of the Ag^+^/gel solutions over different UV irradiation times gradually changed from colorless to yellow, then to brown, and finally dark brown, indicating the formation Ag-NPs in the gelatin solution (Figure 1).

**Figure 2:**
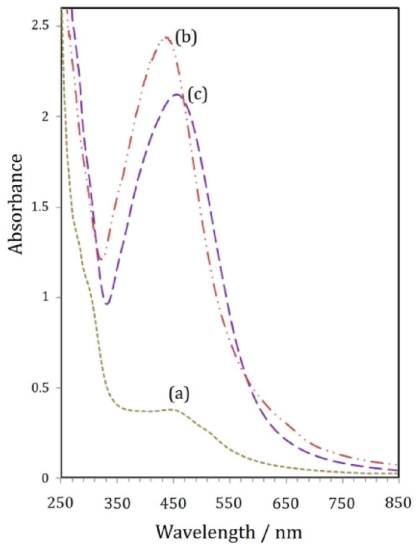
UV-Vis spectra of samples in gelatin solution (1%): (**a**) Ag-NPs prepared without UV-irradiation; (**b**) Ag-NPs UV-irradiated (24 h, Fresh); (**c**) Ag-NPs UV-irradiated (24 h, after 3 month).

The UV-Vis spectroscopy is a reliable route for displaying the presence of metallic nanostructures [21,22]. The role of UV irradiation was verified to define the advance of the silver salt reduction in the presence of gelatin at ambient temperature without UV irradiation, as shown in Figures 2a,b. No large change occurred in the UV-Vis absorption spectrum of the sample prepared without UV irradiation. It was subsequently found that, in the gelatin solution, UV irradiation played a crucial role for the synthesis of Ag-NPs.

In order to investigate the stability of the obtained Ag-NPs, the absorption surface plasmon resonance (SPR) peak of a typical colloidal solution (24 h) was determined for a long period of time (e.g., 3 months). As Figures 2b,c indicate, the position of the SPR peak after 3 months has a small red-shift toward longer wavelengths, with a small decrease in absorbance. This result shows that the colloidal Ag-NPs solutions are stable over a long period of time.

**Figure 3:**
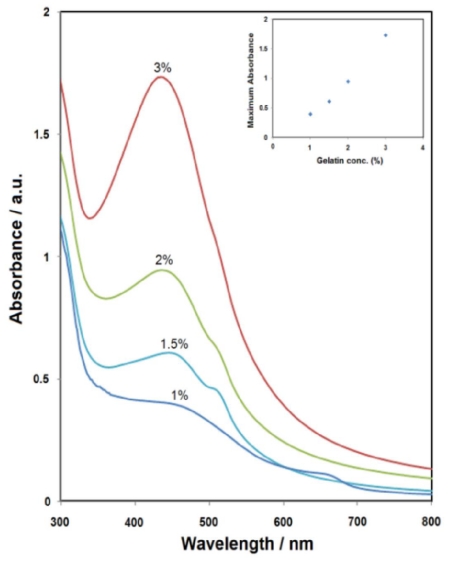
UV-Vis spectra of prepared Ag-NPs at different gelatin concentrations by UV light for 3 h.

To study the effects of gelatin concentrations on Ag-NPs formation, samples with various concentrations of gelatin are prepared and irradiated under UV light for 3 h. When there is 1% gelatin to stabilize Ag-NPs, weak and broad SPR was occurred (Figure 3). As the gelatin concentration is varied from 1.0 to 3.0 wt%, the positions of the SPR bands changed from 445 to 427 nm. Upon increasing the gelatin concentration, however, the intensity is increased remarkably, rendering higher yields of Ag-NPs. It was subsequently found that, in the UV irradiation, gelatin concentration played a crucial role in the synthesis of Ag-NPs.

**Figure 4:**
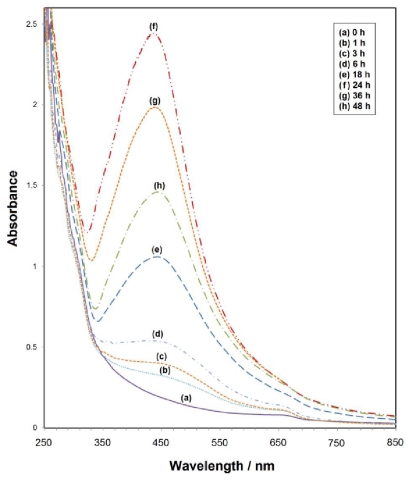
UV-Vis spectra of fabricated Ag-NPs in gelatin solution (1%) under different UV irradiation times.

The UV-Vis spectra of the colloidal solutions were different for various UV irradiation times, although there was no change in the gelatin solution or AgNO_3_ concentration (Figure 4).

As indicated in Figure 4c, after 3 h of UV irradiation, the appearance of a SPR peak at about 468 nm indicated the formation of Ag-NPs [23,24]. From the results obtained from the UV-Vis spectra, the symmetric SPR implies that the size distribution of the Ag-NPs is narrow. The UV-Visible absorption spectrum of Ag-NPs depends on the size of the nanoparticles, indicating that the absorbance at maximum absorption wavelength (λ_max_) increases as the number of nanoparticle increases. Increased particle concentrations are evident with increased UV irradiation times in this case (see Figures 4d–f). Therefore, the UV irradiation of the colloidal solutions for long times at room temperature is sufficient for the preparation of high-concentration solutions of Ag-NPs. At a high UV-irradiation time *i.e.*, 36 and 48 h (4 g and 4 h), the SPR peak is broader while the intensity is lower than 24 h. This result can be attributed to the gelatin degradation under the long-term UV-irradiation source. When many gelatin molecules degrade into the small fragments at a high irradiation time, some Ag-NPs that cannot be enveloped in the gelatin framework agglomerate into larger particles. Thus, the Ag-NPs number decreases and the SPR intensity is decreased because the reduction of the absorbance with the UV-irradiation time indicates that the concentration of Ag-NPs decreases [25].

Based on Mie’s theory [26], nanoparticles with different sizes should demonstrate different optical properties due to the difference in the SPR bands. As shown in Figure 4, the SPR of gelatin stabilized Ag-NPs possessing different diameters. As such, when the particle diameter decreased from 35.8 to 18.8 nm, the λ_max_ of SPR blue shifted from about 461 to 435 nm. The absorption peak due to the SPR of metallic nanoparticles displayed the blue-shift with decreases in the particle diameter [27].

This result demonstrates that the larger Ag-NPs were obtained under shorter UV irradiation time and disintegrated due to further irradiation of UV light. Photoinduced fragmentation of Ag-NPs has been reported [28,29] as following:

(5) $${\left(Ag\right)}_{n}\overset{\text{h}\nu }{\to }{\left(Ag\right)}_{n}^{+}+e_{aq}^{-}$$

(6) $${\left(Ag\right)}_{n}^{+}+e_{aq}^{-}\to {\left(Ag\right)}_{n}$$

(7) $${\left(Ag\right)}_{n}^{+}\to {\left(Ag\right)}_{n-1}+{Ag}^{+}$$

where, (Ag)*_n_* is the silver nanocluster containing n silver atoms and e^−^ _aq_ is the aqueous electron. After UV irradiation to aqueous solutions of gelatin/Ag^+^, a large amount of aqueous electrons were produced, and the silver cations were reduced into Ag-NPs. Therefore, it is possible to control the size and quantity of the Ag-NPs by varying the time of UV irradiation applied to the silver cations solutions. Such a blue-shift is possible because the exposure of UV light to the sample for a long time could slightly change the arrangement around the nanoparticle surface. In addition, the nanoparticle heats a bit and could anneal its surface surrounding leading to a small decreasing in size [30]. Time-dependent phenomena had been reported in synthesis of nanoparticles by UV light [31,32].

**Figure 5:**
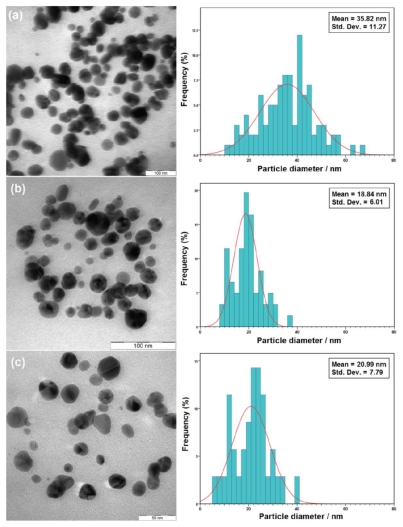
TEM images and corresponding size distributions of fabricated Ag-NPs in gelatin solution (1%) at different UV-irradiation times: (**a**) 6 h; (**b**) 24 h; and (**c**) 48 h.

The TEM images also demonstrate the formation of Ag-NPs under UV-irradiation after 6, 24, and 48 h. Figure 5 shows typical TEM images and the corresponding particle size distribution of the prepared Ag-NPs at different times. The TEM results confirmed the UV-Vis spectra and indicated that the samples obtained over a longer time period (24 h) retained a narrower particle size distribution.

**Figure 6:**
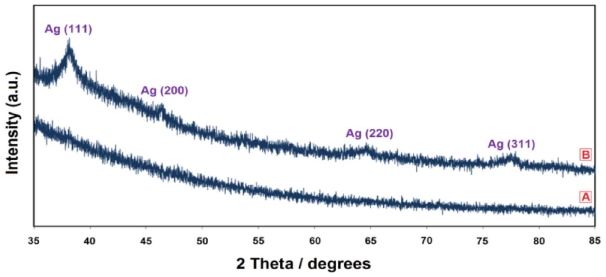
XRD patterns of gelatin/AgNO_3_ (**A**) and gelatin based Ag-NPs after UV irradiation for 24 h; (**B**) in gelatin solution (1%).

The XRD spectrum of the prepared Ag-NPs in the UV irradiated instance for 24 h is shown in Figure 6. The XRD peaks at 2θ degrees of 38.1, 46.3, 64.6, and 77.5 can be attributed to the (111), (200), (220), and (311) crystalline planes of face-centered-cubic (fcc) crystalline structure of metallic silver, respectively (JCPDS file No. 00-004-0783).

All the silver peaks are indexed and indicate that the sample consisted of highly pure Ag-NPs without any impurity. The average crystalline size of the sample UV irradiated for 24 h was calculated from the XRD pattern using the Scherrer formula (Equation 8) [33]:

(8) $$D=\frac{0.94\lambda }{WCos\theta }$$

where, W represents the peak full width at half-maximum intensity of the (111) peak; λ is the wavelength for CuK_α_ (λ = 0.15418 nm); and D is the crystalline size in nanometers. The average crystalline size of the sample (24 h under UV irradiation) was 15.4 nm, which is relatively close to the average size (18.8 nm) of the Ag-NPs as displayed in the TEM image.

**Figure 7:**
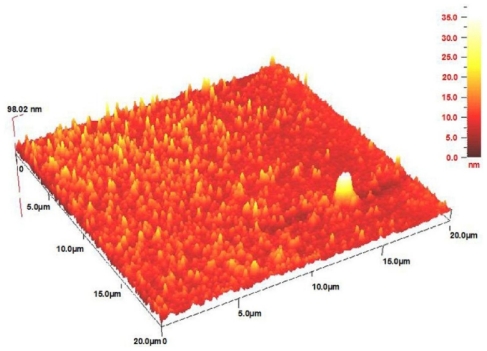
AFM image of gelatin based Ag-NPs after 24 h UV irradiation.

The AFM result displays the surface morphology of the fine-dispersed Ag-NPs formed in the gelatin matrix. As shown in Figure 7, the value determined by the AFM was close to the TEM-determined value, and the films of gelatin containing Ag-NPs displayed a dense and uniformly packed structure. Thus, the Ag-NPs gelatin films could provide a biocompatible and rough surface for special biological applications, such as cell immobilization.

## 3. Experimental Section

### 3.1. Materials

All Chemicals in this work were analytical grade and used as received without further purification. AgNO_3_ (99.98%, Merck KGaA, Darmstadt, Germany), and gelatin (type B, Sigma-Aldrich, St Louis, MO, USA) were used as silver precursor, and stabilizer agent, respectively. All glass wares used in lab experimental were cleaned with freshly solution of HNO_3_/HCl (3:1, v/v), washed thoroughly with doubly distilled water and dried before use.

### 3.2. Synthesis of UV-Irradiated Ag-NPs

For the synthesis of Ag-NPs, 2.0 g gelatin was added to 190 mL H_2_O in a flask, and the solution was stirred to obtain a clear solution. Aqueous AgNO_3_ (10 mL, 1 M) was added to the gelatin solution by continuously stirring to obtain Ag^+^/gel-sol, which was placed into the UV reactor for UV irradiation at different times (*i.e.*, 1, 3, 6, 18, 24, 36, and 48 h) at room temperature. The UV irradiation process was carried out on a UV reactor (UV-A, 6 W).

### 3.3. Characterization Methods and Instruments

The UV irradiated Ag-NPs which prepared under various UV irradiation times were characterized by using ultraviolet-visible (UV-Vis) spectroscopy, transmission electron microscopy (TEM), X-ray diffraction (XRD), and atomic force microscopy (AFM). The optical absorption properties of prepared samples were characterized using a Lambda 35^®^ (PerkinElmer, Waltham, MS, USA) UV-Vis spectrophotometer over the range of 300–700 nm. TEM images were performed using a Hitachi H-7100^®^ electron microscopy (Hitachi High-Technologies Corporation, Tokyo, Japan), and the particle size distributions of nanoparticles were determined using the UTHSCSA Image Tool^®^ Version 3.00 program (UTHSCSA Dental Diagnostic Science, San Antonio, TX, USA). The XRD patterns were carried out on Philips (X’pert, Cu K_α_) and were recorded at a scan speed of 2°/min. The AFM image also was carried out on an Ambios Q-scope^®^ (Ambios Technology, Santa Cruz, CA, USA) (SPM) machine.

## 4. Conclusions

Preparation of Ag-NPs in aqueous gelatin solutions under UV light irradiation without any reducing agent or heat treatment is simply possible. The studies of UV-Vis absorption spectra reveal that the SPR bands of silver nanostructures are clearly affected by the UV irradiation times and gelatin concentrations. The TEM images of Ag-NPs and their particle size distributions indicated that the smaller Ag-NPs were obtained in longer UV irradiation times. The XRD pattern also displays the fcc geometry for obtaining Ag-NPs. These fabricated Ag-NPs are very stable over a long period of time (e.g., 3 months) in an aqueous solution without any sign of agglomeration or precipitants. These results suggest that photoreduction methods such as UV irradiation can maintain Ag-NPs in the presence of green stabilizers (e.g., gelatin at ambient temperature).
